# Age-Related Epigenetic Drift Shapes Coordinated microRNA Promoter Methylation and Expression in Prostate Cancer

**DOI:** 10.3390/epigenomes10020027

**Published:** 2026-04-09

**Authors:** Fernando Bergez-Hernández, Martín Irigoyen-Arredondo, Lizeth Carolina Flores-Méndez, Alejandra Paola Martínez-Camberos

**Affiliations:** Laboratorio de Investigación Biomédica y Biotecnológica, Lic. en Ciencias Biomédicas, Universidad Autónoma de Occidente, Av del Mar 1200, Tellerías, Mazatlán 82100, Mexico; fernando.bergez@uadeo.mx (F.B.-H.); martin.irigoyen@uadeo.mx (M.I.-A.); lizeth.flores@uadeo.mx (L.C.F.-M.)

**Keywords:** prostatic neoplasms, microRNAs, DNA methylation, epigenetic drift, aging

## Abstract

**Background:** Aging is the strongest risk factor for prostate cancer (PCa). It is accompanied by progressive epigenomic divergence, known as epigenetic drift, particularly affecting DNA methylation at regulatory regions. However, the extent to which age-associated promoter methylation contributes to coordinated microRNA (miRNA) expression changes in PCa remains incompletely characterized. **Methods:** We conducted an integrative in silico analysis of 449 primary tumors from the TCGA-PRAD cohort. Age was modeled as a continuous variable. Age-related miRNA expression changes were estimated from miRNA-seq data using DESeq2. Promoter DNA methylation changes (±2 kb from transcription start sites) were assessed using Illumina 450K arrays and linear regression. MiRNAs showing significant age-associated alterations at both expression and methylation levels were classified as concordant or discordant based on directionality and prioritized using an effect size-based concordance score. We analyzed experimentally validated targets of prioritized miRNAs through functional enrichment and network-based approaches to identify convergent regulatory pathways. **Results:** Initially, we identified 105 age-associated miRNAs. After filtering, 65 candidates remained. Of these, we found 37 miRNAs with significant age-associated changes at both layers, including 20 concordant and 17 discordant miRNAs. These comprised well-characterized cancer-associated miRNAs and lesser-studied candidates enriched in CpG-rich regulatory regions. Network analyses revealed a limited set of genes under convergent regulation by multiple age-associated miRNAs. These implicated pathways are related to cell cycle control, apoptosis, stress response, and epigenetic regulation. **Conclusions:** Our findings support a model in which age-dependent promoter methylation drift contributes to coordinated miRNA deregulation in PCa. This convergence highlights biologically plausible miRNA biomarkers and age-sensitive epigenetic circuits relevant to prostate carcinogenesis.

## 1. Introduction

Prostate cancer (PCa) is closely associated with aging. It is one of the leading causes of morbidity and mortality in adult men [1]. In 2022, 1.47 million new cases of PCa were reported globally, with an age-standardized incidence rate of 29.4 per 100,000 men. This makes it one of the most diagnosed cancers in men. About 432,463 deaths from this disease were reported in the same year. In Mexico, an estimated 27,096 cases and 9256 deaths from PCa were reported, with an age-standardized incidence rate of 52.3 in 2019 [2,3]. Although cancer is known to be a multifactorial disease, it has been observed that the incidence and aggressiveness of this type of cancer increase progressively with age, highlighting the importance of understanding the molecular mechanisms that link aging with the development of this carcinogenesis. In Mexico, about six out of ten PCa cases are diagnosed in men aged 65 or older, underscoring the strong association with age [4,5].

One of the molecular modifications associated with age and cancer development is epigenetic drift, a phenomenon characterized by the progressive, age-associated accumulation of epigenetic alterations, such as DNA methylation [6,7]. An age-associated increase in promoter methylation of genes related to normal prostate tissue has been reported, which may precede carcinogenesis and the transition from benign to malignant tissue [6,8,9]. In this context, changes in methylation patterns have been reported in CpG-rich regions, particularly in CpG islands located in gene promoters, functioning as preferential targets for DNA methylation. These regions play a crucial role in the regulation of gene expression through DNA methylation and play a key role in the transcriptional control of miRNAs [10]. Previous studies have identified that under normal conditions, CpG islands tend to remain unmethylated. However, during aging and carcinogenesis, they exhibit aberrant hypermethylation, which can alter miRNA expression, indirectly impacting the regulation of gene expression of tumor suppressor genes involved in DNA repair, cell cycle control, apoptosis, cell adhesion, and signal transduction [9,11,12].

MicroRNAs (miRNAs) are small non-coding RNAs 19–25 nucleotides in length that participate in the regulation of messenger RNA expression and have been shown to have a significant impact on various cellular processes [10]. Their dysregulation has been observed in multiple human neoplasms, including PCa, demonstrating that miRNAs can act as both oncogenes and tumor suppressors [11,13,14]. Recent research has identified miRNAs whose reduced expression is associated with methylation of their promoter regions, demonstrating coordination between methylation and miRNA levels. For example, miR-152-3p, miR-10a, miR-23b, and miR-27b have been identified as having promoter methylation and reduced expression in tumor tissues compared to normal prostate tissue, while miR125b, miR-335-3p, and miR-543 play central roles in cell cycle regulation and DNA repair processes, supporting the idea that epigenetic regulation of miRNAs contributes to tumor progression [14,15,16,17]. Despite this evidence, the contribution of age-associated epigenetic drift to coordinated changes in miRNA expression and DNA methylation in PCa remains incompletely characterized. This study aimed to comprehensively analyze the relationship between age and changes in miRNA expression and DNA methylation in PCa, using public data from the TCGA-PRAD cohort, to identify molecular patterns associated with aging.

## 2. Results

### 2.1. Age-Associated miRNA Expression Changes in Prostate Cancer

**Figure 1:**
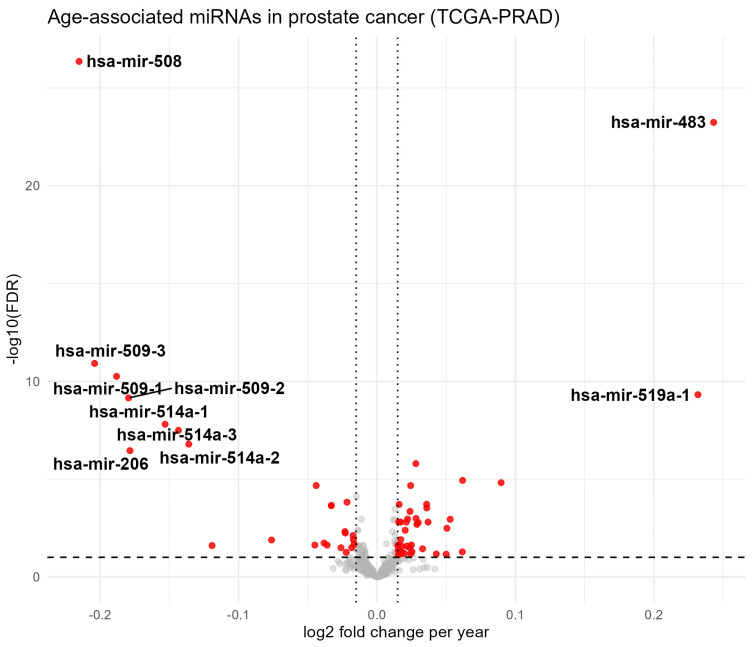
Age-associated miRNAs in prostate cancer. Expression profiles of 65 age-associated miRNAs in PCa are presented, highlighting the 10 miRNAs with the largest age-associated expression changes.

MiRNA expression profiles were analyzed in a total of 449 PCa tumor samples from the TCGA-PRAD cohort with available age information. Patient age ranged from 43.5 to 78.6 years, with a mean age of 61.6 years (median, 62.0 years; interquartile range, 9.8 years). This age distribution provided sufficient variability to model age as a continuous variable in downstream analyses. Differential expression analysis revealed widespread age-associated changes in miRNA expression. Using a false discovery rate (FDR) threshold of 0.10, 55 miRNAs showed increased expression with age, whereas 50 miRNAs exhibited decreased expression with age. To focus on miRNAs with biologically meaningful effect sizes, an additional fold-change filter was applied (log2FC ≥ 0.015, corresponding to approximately a 1% change per year). Applying these combined criteria resulted in a final set of 65 miRNAs whose expression was significantly associated with patient age in PCa tumors (Figure 1).

### 2.2. Age-Associated DNA Methylation Changes at miRNA Promoters

Genome-wide DNA methylation analysis was performed using Illumina HumanMethylation450 array data. A total of 485,577 CpG sites passed quality control and were included in the analysis.

MiRNA promoter regions were defined as ±2 kb around the transcription start site. Using this definition, 4582 CpG sites are mapped to promoter regions of 920 distinct miRNAs, indicating widespread epigenetic coverage of miRNA regulatory regions. CpG-level linear regression models revealed age-associated methylation changes across multiple miRNA promoters, with effect sizes generally in the expected range for age-related epigenetic alterations (on the order of 10^−3^ beta units per year). These effect sizes are consistent with previously reported age-associated DNA methylation changes in human tissues. For each miRNA, promoter methylation was summarized by calculating the mean age-associated methylation coefficient across all associated CpGs, providing an miRNA-level estimate of promoter methylation change with age.

### 2.3. Integrative Analysis Identifies Epigenetically Concordant Age-Associated miRNAs

To investigate whether age-associated changes in miRNA expression were linked to promoter DNA methylation, miRNA expression and methylation datasets were integrated at the miRNA level. After harmonizing miRNA identifiers across platforms, among the 65 age-associated miRNAs, 37 exhibited both significant expression changes and available promoter methylation data and were included in the integrative analysis. MiRNAs were classified based on the directional concordance between promoter methylation and expression changes. Twenty miRNAs (54%) displayed concordant regulation, characterized by promoter hypermethylation associated with decreased expression or promoter hypomethylation associated with increased expression. In contrast, 17 miRNAs (46%) were classified as discordant, showing expression changes inconsistent with the canonical epigenetic regulatory model.

**Figure 2:**
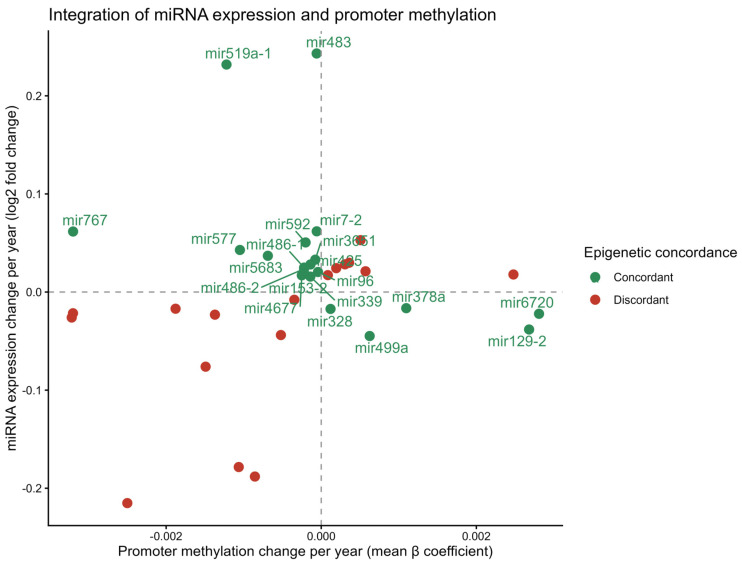
Integrative analysis of age-associated miRNAs. The figure shows promoter methylation versus expression changes, with concordant and discordant miRNAs indicated and top-ranked candidates highlighted.

Scatter plot visualization of promoter methylation versus expression changes revealed that concordant miRNAs clustered predominantly in quadrants consistent with epigenetic repression or derepression, whereas discordant miRNAs were more diffusely distributed (Figure 2). These results suggest that a substantial subset of age-associated miRNA expression changes in PCa may be mediated, at least in part, by promoter DNA methylation. While not all age-associated miRNA expression changes were concordant with promoter methylation, the substantial proportion of concordant miRNAs supports a role for epigenetic regulation in shaping age-related miRNA expression patterns (Table 1).

To assess the robustness of these findings, a sensitivity analysis adjusting for the Gleason score and pathological T stage was performed. Of the 20 concordant miRNAs identified in the primary analysis, 10 remained concordant after adjustment, indicating that a subset of miRNAs is associated with age independently of tumor aggressiveness (Table 1).

### 2.4. Prioritization of Candidate Age-Associated Epigenetic miRNA Biomarkers

To prioritize miRNAs with the strongest evidence of coordinated epigenetic and transcriptional regulation during aging, concordant miRNAs were ranked using a concordance score that integrates the magnitude of age-associated changes in expression and promoter methylation. The concordance score was defined as the product of the absolute value of the regression coefficient for miRNA expression change per year of age and the absolute value of the regression coefficient for promoter methylation change per year of age. For each concordant miRNA, both the percentage change in expression and promoter methylation per year were estimated, allowing direct biological interpretation of age-related effects. These miRNAs showed either progressive upregulation accompanied by promoter hypomethylation or gradual downregulation associated with promoter hypermethylation (Table 1). MiRNAs were further ranked based on the concordance score integrating expression and methylation trends. This multiplicative approach prioritizes miRNAs showing strong age-associated effects at both the transcriptional and epigenetic levels, while down-weighting miRNAs with large changes in only one molecular layer. Several of the top-ranked concordant miRNAs have been previously implicated in cancer-related processes, supporting the biological relevance of this integrative ranking approach.

**Table 1:** Integrated expression and promoter methylation changes of age-associated concordant miRNAs in prostate cancer in the TGCA cohort.

Rank	miRNA	Expression		Methylation		Conc. Score
		%/year	Direction	%/year	Status	
1	miR-519a-1	+0.174	Up	−0.122	Hypo	2.489
2	miR-129-2 *	−2.612	Down	+0.268	Hyper	2.102
3	miR-6720	−1.525	Down	+0.281	Hyper	1.763
4	miR-767 *	+4.36	Up	−0.32	Hypo	0.687
5	miR-378a	−1.134	Down	+0.11	Hyper	0.635
6	miR-499a *	−3.059	Down	+0.062	Hyper	0.545
7	miR-328	−1.187	Down	+0.012	Hyper	0.082
8	miR-483 *	+18.35	Up	−0.006	Hypo	0.079
9	miR-4677	+1.189	Up	−0.025	Hypo	0.054
10	miR-153-2 *	+1.387	Up	−0.022	Hypo	0.042
11	miR-486-2	+1.652	Up	−0.022	Hypo	0.034
12	miR-486-1	+1.741	Up	−0.022	Hypo	0.031
13	miR-339 *	+1.098	Up	−0.014	Hypo	0.029
14	miR-577	+3.017	Up	−0.105	Hypo	0.025
15	miR-5683	+2.595	Up	−0.069	Hypo	0.024
16	miR-592	+3.566	Up	−0.02	Hypo	0.018
17	miR-425 *	+1.967	Up	−0.014	Hypo	0.015
18	miR-7-2 *	+4.383	Up	−0.006	Hypo	0.008
19	miR-3651 *	+2.312	Up	−0.008	Hypo	0.004
20	miR-96 *	+1.426	Up	−0.004	Hypo	0.004

Expression (%/year): estimated age-associated percentage change in miRNA expression per year. Methylation (%/year): estimated age-associated percentage change in promoter DNA methylation per year. Concordant score (Conc. score): product of the absolute expression and methylation coefficients. * MiRNAs age-associated after adjustment for Gleason score and pathological T stage.

### 2.5. External Validation of Age-Associated miRNAs

To assess reproducibility, candidate miRNAs were evaluated in the independent GSE135535 cohort. Several miRNAs, including miR-129-2, miR-6720, miR-767, miR-483, miR-339, miR-577, and miR-592, showed consistent directionality of age-associated expression changes with the TCGA cohort. In contrast, other miRNAs displayed discordant patterns, suggesting potential context-dependent regulation. Overall, these findings indicate partial reproducibility of age-associated miRNA expression changes across independent datasets (Table 2). For DNA methylation, the miRNAs were evaluable in the independent GSE127985 cohort. Among these, 10 (50%) showed concordant directionality of age-associated methylation changes compared to TCGA, while three (15%) showed discordant results (Table 1), supporting overall consistency of epigenetic alterations across cohorts. Not all candidate miRNAs were evaluable in the cohort validation due to platform-specific coverage and annotation limitations; those lacking sufficient data were assigned NA. In the expression dataset, certain miRNAs were not included in the NanoString panel, while in the methylation dataset, some miRNA loci lacked sufficient CpG coverage within promoter regions to enable reliable analysis. Given the limited sample size and the small, expected effect sizes characteristic of age-associated epigenetic changes, validation was primarily assessed based on the directionality of these trends rather than statistical significance alone. The high proportion of concordant directionality supports the robustness of these age-associated patterns across independent datasets.

### 2.6. Functional Convergence of Age-Associated miRNAs

**Figure 3:**
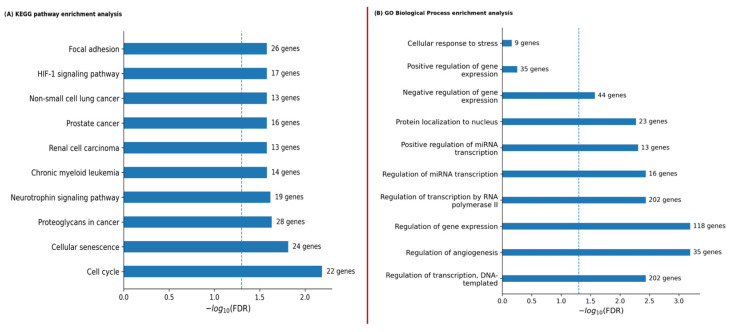
KEGG and Gene Ontology enrichment analysis of age-associated miRNA target genes. (**A**) KEGG pathways and (**B**) GO Biological Processes significantly enriched among predicted miRNA targets. Bar length corresponds to −log_10_(FDR), the dashed line marks the significance threshold (FDR = 0.05), and gene counts are shown for each term.

Experimentally validated targets of age-associated miRNAs were retrieved using miRTarBase via miRNet 2.0, yielding 1476 genes for functional analysis. KEGG enrichment revealed a marked convergence toward pathways related to aging and cancer, including cell cycle regulation, cellular senescence, proteoglycans in cancer, neurotrophin signaling, and PCa-associated pathways (Figure 3A). Gene Ontology Biological Process analysis consistently highlighted transcriptional regulation, cell cycle control, and processes related to cellular aging and proliferation (Figure 3B). Collectively, these findings indicate that age-associated miRNAs coordinately regulate core pathways, linking aging-related mechanisms with oncogenic signaling in PCa.

### 2.7. Network-Based Identification of Convergent Central Regulatory Genes

The miRNA–gene interaction dataset was analyzed using a network-based approach with a degree threshold (degree ≥ 5), resulting in a reduced set of genes under multi-miRNA regulation. Within this filtered network, genes such as MDM2, SOD2, XIAP, RAN, and ZZZ3 were retained based on their connectivity and are associated with processes including apoptosis, oxidative stress response, and cell cycle regulation, consistent with Gene Ontology and KEGG enrichment analyses. These genes and their associated miRNAs are summarized in Table 3.

**Figure 4:**
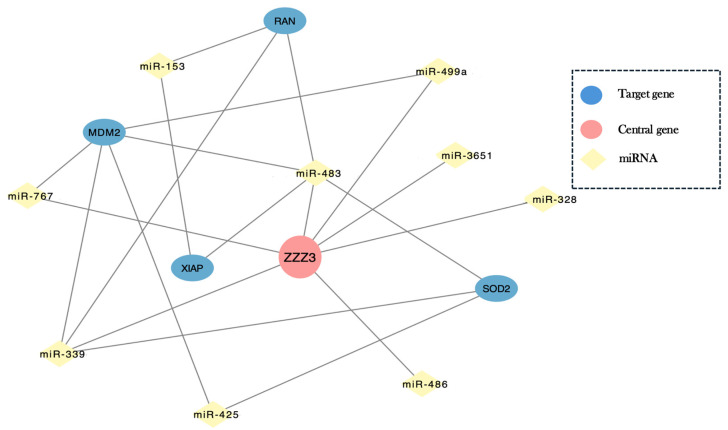
Network representation of convergent regulation by age-associated miRNAs in prostate cancer. The network shows experimentally validated miRNA–gene interactions obtained from miRTarBase and visualized in Cytoscape after degree-based filtering (genes targeted by ≥5 miRNAs). Nodes represent genes (blue) and miRNAs (light yellow). ZZZ3 is shown in red due to its higher connectivity within the network. Edges represent validated interactions. The filtered network highlights genes under multi-miRNA regulation.

In this context, ZZZ3 showed the highest degree within the network and was connected to multiple genes and miRNAs. Among these, miRNAs such as miR-153, miR-483, miR-3651, miR-486, and miR-339 were identified as directly interacting with ZZZ3. Overall, the filtered network displayed a reduced structure in which multiple miRNAs and genes remained connected within the same module (Figure 4).

## 3. Discussion

Epigenetic drift refers to age-related DNA methylation changes and the tendency for increasing discordance between epigenomes over time, leading to increased interindividual variability in gene regulation [7]. In age-associated malignancies such as PCa, this progressive epigenetic drift contributes to tumor initiation, progression, and molecular heterogeneity [6]. The progressive alterations in DNA methylation patterns, both globally and in specific regions, can affect the expression of miRNAs involved in maintaining prostate homeostasis and tumor biology [18,19]. However, these changes do not uniformly affect all miRNAs, since their epigenetic regulation depends on both their genomic localization and the cellular context. Together, these observations provide a framework to explore coordinated alterations in miRNA expression and promoter methylation in PCa and supports their relevance as biomarkers associated with age and tumor risk.

Our integrative analysis provides evidence that age-associated epigenetic drift in PCa is not a random process but instead manifests as coordinated remodeling of miRNA promoter methylation and expression. By modeling age as a continuous variable, we identified consistent, gradual changes across both molecular layers, supporting a model in which aging progressively reshapes the miRNA regulatory landscape. The initial analysis of the 20 miRNA candidates from the TGCA-PRAD cohort exhibited concordant methylation–expression patterns, indicating that promoter methylation contributes to the directionality of miRNA deregulation. In addition, cross-cohort validation on GEO datasets revealed partial but consistent reproducibility, with several miRNAs maintaining the directionality of expression and/or methylation changes across independent datasets; a group of eight and ten miRNAs maintained concordant patterns on expression and methylation, respectively. Rather than complete overlap, this pattern of partial concordance is expected given biological heterogeneity, platform differences, and the modest effect sizes characteristic of age-associated alterations. Together, these results support the existence of a robust core of age-associated miRNAs, while also highlighting the context-dependent nature of epigenetic regulation in PCa [18,19].

Within this framework, a reduced set of miRNAs emerged as the most robust candidates integrating evidence from expression, promoter methylation, and cross-cohort validation. miR-129-2, miR-153-2, and miR-339 consistently exhibited concordant age-associated changes across datasets and remained stable after adjustment for clinical covariates, supporting their potential role as age-sensitive epigenetic regulators in PCa. miR-129-2 constitutes a well-established example of epigenetically regulated miRNA silencing across multiple epithelial cancers. Its promoter hypermethylation has been consistently linked to decreased expression and enhanced tumor cell proliferation in breast cancer, hepatocellular carcinoma, and other epithelial tumors, supporting a conserved methylation-dependent regulatory mechanism [20,21]. Similarly, miR-153 has been reported to be highly expressed in PCa and to directly target and downregulate PTEN, leading to activated AKT signaling [22]. Its upregulation in PCa is often associated with the overall loss of methylation control [23]. Another study shows that miR-339 is capable of controlling DNA methylation changes through targeting DNMT3B in colon cancer cell lines, where increased levels of these miRNA effectively resulted in DNMT3B downregulation [24]. Collectively, these miRNAs displayed coordinated patterns of promoter methylation and expression consistent with canonical epigenetic regulation, suggesting, in our study, a direct contribution of aging on promoting structured and biologically meaningful regulatory shifts that converge on post-transcriptional control mechanisms in prostate carcinogenesis [7,19].

Additionally, miR-592 showed consistent concordant behavior and validation across datasets but did not retain significance after covariate adjustment, indicating that its regulation may be partially influenced by tumor-related factors. This context-dependent pattern aligns with previous reports describing miR-592 as both upregulated and downregulated in PCa depending on disease stage and experimental context [25,26,27,28], with functional evidence linking its upregulation to increased proliferation via FOXO3a targeting [27]. Overall, these findings support miR-592 as a biologically relevant, yet context-dependent, candidate in age-associated miRNA regulation. Together, these miRNAs represent a prioritized subset with strong integrative evidence, providing biologically plausible links between aging, epigenetic regulation, and miRNA-mediated control in PCa.

In line with these observations, gradual accumulation of both DNA hypomethylation and locus-specific hypermethylation events increased with cancer patient age, in contrast with the age-independent genomic alterations [29]. Given the inherent versatility of miRNA-mediated regulation, where single miRNAs can target multiple genes and individual genes can be regulated by multiple miRNAs, this system is vulnerable to dysregulation when miRNA expression levels are altered during aging [30]. Our findings align with and extend this paradigm by identifying coordinated changes in miRNA expression and promoter methylation associated with age in PCa.

Notably, several miRNAs identified in our TCGA-derived candidate set have been previously described as biologically relevant examples of methylation-mediated regulation. For instance, miR-378a has been shown to be downregulated through DNMT1-mediated promoter hypermethylation in hepatocellular carcinoma, linking epigenetic silencing to oncogenic pathways such as NF-κB signaling [31,32,33]. Similarly, miR-483 exemplifies an indirect mechanism of epigenetic control, as its expression is tightly coupled to the methylation status of its host gene, IGF2, where promoter hypomethylation drives coordinated overexpression and oncogenic activity [34,35,36]. These models support the notion that age-related methylation changes may regulate miRNA expression through both direct promoter effects and broader genomic context. Moreover, many miRNAs do not possess independent canonical promoters but instead depend on CpG-rich regions within host genes or shared regulatory loci, making them particularly sensitive to age-related methylation drift [18,19], reinforcing the biological plausibility of the coordinated patterns observed in our analysis.

In addition, the results revealed the identification of miRNAs with discordant patterns between promoter methylation and expression. This suggests that miRNA regulation is influenced by multiple regulatory factors and not solely by DNA methylation [37]. In this context, there are indirect regulatory processes mediated by transcription factors that could modulate miRNA expression independently of local methylation status [38]. Moreover, the activity of distal regulatory elements, such as enhancers, can contribute to the activation or repression of miRNAs independently of their promoters [39]. Furthermore, modifications in histone marks represent a key mechanism that can generate active or repressive chromatin states, explaining discrepancies between methylation and expression [38,40]. In the case of miR-34a, for example, it has been shown that its silencing may involve not only DNA methylation but also the action of repressive complexes such as PRC2 and histone deacetylases [41,42]. Finally, factors such as the tumor context, including inflammatory signals, interaction with the microenvironment, and cellular heterogeneity, can significantly alter regulatory patterns. In this regard, discordant miRNAs may reflect a more complex and dynamic epigenetic network that exhibits sensitivity to age-related changes [37,43]. These observations suggest that the integration of age-associated epigenetic changes and miRNA-mediated regulation may contribute to the progressive remodeling of regulatory networks in PCa, potentially influencing tumor behavior and heterogeneity. This is particularly relevant in aging-associated malignancies, where cumulative molecular alterations may shape disease progression in a gradual and context-dependent manner.

However, even miRNAs exhibiting concordant methylation–expression patterns may be subject to additional layers of regulation. A network analysis derived from miRNAs showing concordant methylation–expression patterns provided insight into the regulatory architecture underlying these coordinated changes. This analysis suggests that age-associated miRNA alterations may not act independently but instead converge on specific regulatory nodes. Within the network identified in this study, ZZZ3 emerged as the most connected node, indicating a potential point of convergence for multiple miRNAs. ZZZ3 is a core component of the ATAC histone acetyltransferase complex involved in promoter acetylation and transcriptional regulation, suggesting a potential link between miRNA-mediated regulation and chromatin-based epigenetic control mechanisms beyond DNA methylation [44,45]. The convergence of age-associated miRNAs on a chromatin-related regulator such as ZZZ3 suggests that even miRNAs exhibiting canonical methylation–expression relationships may be embedded within broader epigenetic regulatory frameworks. Although the precise role of ZZZ3 in this context remains to be elucidated, its position within the network supports its potential involvement in integrating multiple layers of epigenetic and post-transcriptional regulation.

These findings support a multi-layered regulatory model in which aging-associated epigenetic drift and miRNA deregulation interact to shape gene regulatory networks in PCa. Taken together, these findings highlight the complexity of epigenetic regulation in aging. In this context, it is important to consider certain aspects and limitations. Validation was performed on independent GEO cohorts, where differences in sample size, data generation platforms (miRNA-seq in TCGA vs. NanoString in GSE135535), and coverage of miRNAs and CpG sites constitute expected sources of variability in integrative genomic analyses. These conditions may influence the detection and magnitude of associations, particularly considering that age-related changes exhibit modest effect sizes. Furthermore, differences between platforms and batch effects are widely recognized factors that limit direct comparability between cohorts [46,47]. In this regard, validation focused on the consistency of the directionality of the changes rather than on statistical significance, an approach well-suited for subtle biological signals in heterogeneous contexts. Additionally, the lack of cohorts integrating multiple molecular layers limited validation to independent datasets, which is a common constraint in multi-omics studies [48]. Importantly, the definition of miRNA promoter regions using a fixed ±2 kb window may not fully capture regulatory elements, particularly for intronic miRNAs that are co-regulated with their host genes. Despite this, the observed concordance in directionality supports the robustness and biological relevance of the identified patterns.

In PCa, an age-associated disease with pronounced epigenetic remodeling, such coordinated regulation may contribute to inter-individual variability in tumor aggressiveness and clinical outcome. Our results support a model in which age-related promoter methylation changes progressively reshape the miRNA landscape, selectively silencing tumor-suppressive miRNAs while permitting the maintenance or upregulation of oncogenic miRNAs.

## 4. Materials and Methods

### 4.1. Data Sources and Study Design

An in silico integrative analysis was performed using publicly available data from The Cancer Genome Atlas Prostate Adenocarcinoma cohort (TCGA-PRAD). MiRNA expression (miRNA-seq), DNA methylation (Illumina HumanMethylation450), and clinical data were retrieved from the Genomic Data Commons using the TCGAbiolinks package (v2.36.0). Only primary prostate tumor samples with available age information were included. Age was modeled as the primary variable of interest, and it was modeled as a continuous variable in all analyses. Additional clinical covariates were not included to specifically capture global age-associated molecular trends. Independent validation of age-associated miRNAs was performed using datasets obtained from the Gene Expression Omnibus (GEO) database.

### 4.2. Harmonization of miRNA Identifiers and Methylation–miRNA Mapping

To enable integration between miRNA expression and DNA methylation datasets, miRNA identifiers were harmonized to a common base format by removing species prefixes (e.g., hsa-), arm annotations (-5p/-3p), and hyphen separators, generating standardized identifiers corresponding to the core miRNA gene name (e.g., hsa-miR-129-2-3p → mir1292).

DNA methylation data from the TCGA Illumina HumanMethylation450K platform were mapped to miRNA loci using probe annotations from the array manifest. CpG probes were associated with miRNA regions based on genomic annotations, and when multiple probes mapped to a given miRNA locus, summary statistics were calculated across the associated CpGs prior to integration with expression data.

### 4.3. miRNA Expression Analysis

Raw miRNA read counts were processed using TCGAbiolinks and analyzed with DESeq2 (v1.48.2). Less expressed miRNAs were filtered prior to analysis. Differential expression was modeled as a function of age, with age values mean-centered to improve model convergence. Log2 fold changes represent estimated expression changes per year of age. Statistical significance was assessed using Wald tests, and multiple testing corrections were applied using the Benjamini–Hochberg false discovery rate (FDR). An initial exploratory analysis using an FDR threshold of 0.10 identified candidate miRNAs, followed by a more stringent filtering step (FDR ≤ 0.015 and a minimum effect size threshold) to define the final set used for downstream analyses.

### 4.4. DNA Methylation Analysis

Genome-wide DNA methylation beta values were processed and quality-controlled using the sesame framework (v1.26.0). CpG-level age-associated methylation changes were assessed using linear regression models, with beta values modeled as a function of age. Resulting *p*-values were adjusted for multiple testing using the Benjamini–Hochberg procedure.

### 4.5. miRNA Promoter Annotation and Methylation Summarization

MiRNA genomic coordinates were obtained from Ensembl using AnnotationHub and GenomicRanges. Promoter regions were defined as ±2 kb around the transcription start site. CpG probes were mapped to miRNA promoter regions using genomic annotations from the Illumina HumanMethylation450K array manifest. For each miRNA, promoter methylation was summarized as the mean age-associated methylation coefficient across all promoter-associated CpGs, and the minimum adjusted *p*-value was retained.

### 4.6. Integration of Expression and Methylation Data

MiRNA identifiers from expression and methylation datasets were harmonized to a common base format to enable integration. MiRNAs with both age-associated expression and promoter methylation data were integrated at the miRNA level. When multiple annotations mapped to the same miRNA, signals were summarized to avoid redundancy. MiRNAs were classified as concordant when promoter methylation changes were directionally consistent with expression changes (hypermethylation with reduced expression or hypomethylation with increased expression), and as discordant otherwise.

A sensitivity analysis was conducted by including Gleason score and pathological stage as covariates in the regression models to evaluate whether age-associated miRNA changes were independent of tumor aggressiveness. Subsequently, the integration with promoter methylation data was repeated using the adjusted results to identify concordant miRNAs that remained significantly associated with age after accounting for clinical factors.

### 4.7. Prioritization of Candidate miRNAs

Concordant miRNAs were ranked using a concordance score integrating effect sizes from both molecular layers, defined as the product of the absolute age-associated expression and promoter methylation coefficients. The concordance score was calculated using z-score normalization of both expression and methylation coefficients prior to integration. MiRNAs with the highest scores were prioritized as candidates for epigenetic biomarkers associated with aging in PCa. Also, for interpretability, log2 fold-change and methylation coefficients were converted to approximate percentage changes per year.

### 4.8. External Validation Cohort

Independent validation of age-associated miRNAs was performed using the GSE135535 cohort, comprising 320 primary prostate tumors profiled by NanoString. Expression data and clinical variables were obtained from GEO. For each candidate miRNA, linear regression models were used to assess associations between expression levels and age. Directionality of age-associated expression changes was compared with results from the TCGA-PRAD cohort. DNA methylation validation was performed using an independent GEO cohort (GSE127985). For each miRNA, promoter regions were approximated as ±2 kb around CpG sites annotated to the corresponding miRNA locus using Illumina 450k annotation. Age-associated methylation changes were evaluated using linear regression models, and directionality was compared with TCGA results.

### 4.9. Target Gene Identification and Network Analysis

Experimentally validated target genes of selected age-associated miRNAs were obtained from miRTarBase, retrieved using miRNet 2.0, considering only interactions supported by reporter assays, Western blot, or qPCR. The complete interaction dataset was used for KEGG pathway and Gene Ontology Biological Process enrichment analyses in Enrichr. For network visualization, interactions were imported into Cytoscape (v3.10.4). A stringent degree-based filtering approach was applied, retaining only genes targeted by ≥3 miRNAs nodes with degree ≥5 to identify convergent highly connected regulatory hubs; this was the only filtering applied. Additional nodes were retained to preserve the overall network structure and connectivity. Isolated nodes generated after filtering were subsequently removed to refine the network topology. This filtering was applied exclusively for network analysis and hub identification, while the complete dataset was preserved for enrichment analyses.

### 4.10. Data Visualization and Computational Environment

Data visualization was performed using ggplot2, ggrepel, and pheatmap. All analyses were conducted in R version 4.5.1 (13 June 2025) on a Windows operating system, using DESeq2 v1.48.2, TCGAbiolinks v2.36.0, and sesame v1.26.0.

## 5. Conclusions

This study demonstrates that a substantial subset of PCa miRNAs undergoes age-dependent coordinated remodeling of promoter DNA methylation and gene expression, consistent with epigenetic drift–mediated regulation. Notably, a proportion of these miRNAs (miR-129-2, miR-153-2, and miR-339) remained robust after adjustment for clinical covariates, supporting their association with aging independently of tumor aggressiveness, while others may reflect context-dependent influences. Beyond individual miRNAs, network-based analysis revealed that age-associated miRNA deregulation converges on a restricted set of regulatory genes involved in cell cycle control, apoptosis, stress response, and epigenetic regulation, supporting a systems-level organization of aging-related post-transcriptional regulation.

Collectively, these findings extend the current understanding of miRNA regulation in age-associated malignancies by supporting that chronological aging contributes to selective epigenetic modulation of miRNA promoters in PCa tumors and coordinated regulation of key biological pathways. Although further experimental validation is required, this integrative in silico framework identifies biologically plausible miRNA biomarkers and regulatory circuits susceptible to age-related epigenetic drift, providing a foundation for future mechanistic and translational exploration in prostate carcinogenesis.

## Figures and Tables

**Table 2:** Validation set on GEO databases and concordance with TGCA-PRAD.

Rank	miRNA	Expression		Methylation	
		Direction	Concordance	Status	Concordance
1	miR-519a-1	NA	No	Hypo	Yes
2	miR-129-2 *	Down	Yes	Hyper	Yes
3	miR-6720	Down	Yes	NA	No
4	miR-767	Up	Yes	NA	No
5	miR-378a	NA	No	NA	No
6	miR-499a	Up	No	NA	No
7	miR-328	Up	No	Hypo	No
8	miR-483	Up	Yes	Hyper	No
9	miR-4677	NA	No	NA	No
10	miR-153-2 *	Up	Yes	Hypo	Yes
11	miR-486-2	Down	No	Hypo	Yes
12	miR-486-1	Down	No	Hypo	Yes
13	miR-339 *	Up	Yes	Hypo	Yes
14	miR-577	Up	Yes	Hyper	No
15	miR-5683	NA	No	NA	No
16	miR-592 *	Up	Yes	Hypo	Yes
17	miR-425	Down	No	Hypo	Yes
18	miR-7-2	Down	No	Hypo	Yes
19	miR-3651	NA	No	NA	No
20	miR-96	Down	No	Hyper	Yes

* Concordant miRNAs in TGCA and GEO datasets; NA: miRNAs not identified in validation datasets.

**Table 3:** Genes regulated by age-associated miRNAs in the filtered network and their functional involvement.

Target Gene	No. of miRNA	Regulating miRNAs	Functional Involvement (KEGG/GO)
*ZZZ3*	7	miR-499a, miR-3651, miR-328, miR-486, miR-339, miR-767, miR-483	Transcriptional regulation, chromatin organization
*MDM2*	4	miR-767, miR-483, miR-339, miR-425	p53 regulation, apoptosis, cell cycle control
*SOD2*	3	miR-339, miR-425, miR-483	Oxidative stress response, mitochondrial function
*RAN*	3	miR-153, miR-483, miR-339	Nuclear transport, cell cycle regulation
*XIAP*	2	miR-483, miR153	Apoptosis inhibition, cell survival

## Data Availability

All data analyzed in this study are derived from publicly available datasets. The processed data supporting the findings of this study are included within the article and its Supplementary Materials.

## Supplementary Materials

The following supporting information can be downloaded at: https://www.mdpi.com/article/10.3390/epigenomes10020027/s1, Table S1. 20 Concordant miRNAs expression and methylation; Table S2. 65 MiRNAs age-associated on prostate cancer; Table S3. Metadata of 449 samples; Table S4. Cohort metadata validation expression; Table S5. Cohort metadata validation methylation; Table S6. Final miRNAs with adjustment for covariates; Table S7. miRNA expression validation set; Table S8. miRNA methylation validation set.

## Abbreviations

PCa	Prostate cancer
miRNA	microRNA
FDR	False discovery rate
TCGA	The Cancer Genome Atlas
TSS	Transcription start site
CpG	Cytosine–phosphate–guanine
GO	Gene Ontology
KEGG	Kyoto Encyclopedia of Genes and Genomes
VST	Variance-stabilizing transformation
